# Post-lumpectomy radiation therapy boost in breast cancer patients: evidence revisited

**DOI:** 10.3332/ecancer.2021.1194

**Published:** 2021-03-01

**Authors:** Majd Kayali, Joseph Abi Jaoude, Paul Ramia, Hazem Assi, Fady Geara, Philip Poortmans, Youssef H Zeidan

**Affiliations:** 1 Department of Radiation Oncology, American University of Beirut Medical Center, Beirut, Lebanon; 2 Faculty of Medicine, American University of Beirut, Beirut, Lebanon; 3 Department of Internal Medicine, American University of Beirut Medical Center, Beirut, Lebanon; 4 Department of Radiation Oncology, Institut Curie, Paris Sciences & Lettres, PSL University, Paris, France; † Co-first authors with equal contribution.

**Keywords:** radiation therapy, breast cancer, radiation boost, breast conserving therapy, primary tumour bed

## Abstract

**Purpose:**

Radiation therapy is an integral part in the management of breast cancer after breast conservative surgery. In selected patients at high risk for local recurrence (LR), a boost radiation dose is commonly applied to the tumour bed.

**Methods:**

We performed a review of the English literature using PubMed, Medline and Google Scholar for published manuscripts addressing the effect of boost radiation in breast cancer patients, focusing mainly on LR and overall survival (OS).

**Results:**

A total of seven studies were included in our review. Most studies (6/7, 85.7%) showed a significant improvement in local control independent of age (hazard ratios ranging between 0.34 and 0.73), with the largest absolute benefit in younger patients. None of the studies, however, was able to demonstrate an improvement in OS.

**Conclusions:**

With lack of sufficient studies addressing the role of boost radiation, individualised treatment decisions are recommended, taking into account the risk factors for LR, including tumour biology. Real-life data are sorely needed to better assess the role of tumour bed boost in the contemporary era.

## Introduction

Radiation therapy is a commonly employed adjuvant treatment for breast cancer disease after breast conservative surgery in order to reduce the risk of locoregional recurrence (LRR) and improve survival [1, 2]. Several high-risk features for recurrence have been identified including: extensive nodal involvement, young age, lymphovascular invasion, high mitotic index, hormone receptor negative tumours, large primary tumour size and positive surgical margins [3–6]. Radiation is often administered over the entire breast and draining lymph nodes (LN) with an optional surgical cavity boost for early breast cancer disease after breast-conserving surgery (BCS). This has shown comparable overall survival (OS) and LRR rates to treatment with mastectomy [7, 8]. The concept of tumour bed boost originated from the observation that the majority of ipsilateral breast cancer recurrences developed within close proximity to the original lesion [9]. Thus, a radiation boost is often employed to the tumour bed, with the rationale of eliminating any postsurgical microscopic tumour cells that may lead to local recurrence (LR) in the future [10].

International guidelines recommend a boost dose to selected patients bearing high risk features of LR. Previous research has provided a variety of radiation therapy regimens (doses, fractionation and schedules); however, no consensus was reached on specific boost indications. For instance, National Comprehensive Cancer Network (NCCN) guidelines state that administration of a boost dose is to be considered for patients with higher risk of recurrence [11]. However, with the lack of sufficient high-level evidence, much is left to subjective assessment of the clinician and patient decision. While boost therapy benefits are clear in high-risk patients [12], the benefits are less pronounced in patients with lower risk profiles. With the inconvenience of longer treatment time when delivering sequential radiation therapy, increased cost and increased risk of radiotherapy side effects, cavity boost radiation administration remains a controversial topic. We perform a narrative review of the literature to identify available studies that assessed the role of boost in breast cancer patients.

## Methods

### Search strategy and inclusion/exclusion criteria

In this review, we searched PubMed, Medline and Google Scholar for material in English from the inception of the database until 18 December 2019. We screened studies that analysed patients with non-metastatic breast cancer treated with BCS. We included studies that analysed the effect of radiation therapy boost versus no radiation therapy boost. The main outcomes that were needed for inclusion were LR, ipsilateral breast tumour recurrence or OS. All studies analysing the effect of boost were included, regardless of breast cancer type, stage and grade, and regardless of surgical and/or medical treatment receipt. We included randomised and non-randomised trials, as well as retrospective studies in our review. Unpublished material, abstracts and ongoing trials were excluded from our study.

### Data analysis

All data were manually extracted from the included studies and inserted into spreadsheets. Table 1 summarises the characteristics of our included studies. Total number of patients, number of patients in the boost/no-boost groups, median follow-up, study inclusion criteria, surgery and systematic therapy use were all recorded. For the outcomes, percentages, relative risk (RR) and hazard ratios (HRs) with corresponding *p-values* were extracted when available. Also, adjusted models or propensity-matched models were used when possible. Finally, we performed a narrative review of the literature, and each study was analysed in the context of its population characteristics, Forest plots were generated using Prism version 8.

## Results

A total of seven studies met our inclusion criteria and were included in the current analysis. Results from the majority of studies demonstrated a positive effect of boost in improving local control, with three studies showing statistically significant improvement [13–15]. The radiation doses administered in those studies were not uniform and included 26 Gy [16], 16 Gy [13, 14] and 10 Gy [10].

**Figure 1.**
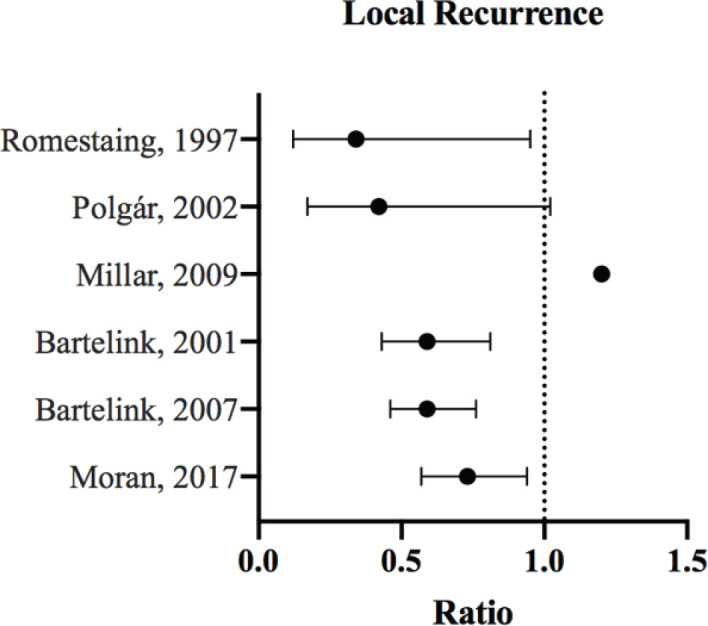
LR ratios and CI for the comparison of radiation therapy boost to no-boost. Bartelink *et al* [13, 33] analysed the same trial population at different follow-up periods, and both reported HRs with 99% CI. Romestaing *et al* [10] and Polgár *et al* [14] reported RR with 95% CI. Millar *et al* [42] reported HR; the *p*-value was 0.599. Moran *et al* [34] reported the HR and corresponding 95% CI for ipsilateral breast tumour recurrence.

Most notably, between 1989 and 1996, the European Organisation for Research and Treatment of Cancer (EORTC) clinical trial recruited 5,569 patients, of whom 5,318 had a microscopical complete excision of the primary breast tumour along with axillary dissection, and who were subsequently randomised to receive a boost dose to the original tumour bed. Patients were followed up to a median of 17.2 years, and results showed a significant improvement in local control (HR = 0.65; 99% confidence intervals (CI), 0.52 to 0.81) with no difference between both arms in terms of breast cancer mortality (*p* = 0.323) (Figure 1) [12]. Additionally, the study showed the highest absolute risk reduction of LR to be in younger patients (age < 40), where the risk was reduced from 36.0% to 24.4% (ARR: 11.6%, HR: 0.56, *p*: 0.003). However, the cumulative incidence of severe fibrosis was found to be significantly higher with boost therapy among age groups >40 (*p* < 0.001) [12]. Within the EORTC patient population, 251 patients with involvement of surgical margins were randomised to receive either a low boost (10 Gy) or high boost dose (26 Gy). After a follow-up period of 10 years, there was no statistical difference in local control or survival between boost doses, with significantly higher incidence of fibrosis in the higher boost dose group [16].

The Lyon trial, published in 1997, analysed the effect of a 10-Gy radiation boost in the treatment of early breast cancer. The study enrolled 1,024 patients with breast cancer treated by local excision, axillary dissection and subsequent whole breast irradiation with/without boost radiation. Significant improvement was seen in local control (RR: 0.3, CI: 0.12–0.95). The boost group had a higher incidence of grade 1 and 2 telangiectasia (12.4% versus 5.9%). However, no significant difference was seen in the self-assessment scoring for cosmetic results [10].

Similarly, the results of the Budapest trial, published in 2002, after 5 years of follow-up demonstrated the importance of boost therapy in local control. Two hundred and seven women were randomised, after administration of whole breast irradiation, to receive either a boost to the tumour bed or no further radiotherapy. Local tumour control was better in the boost group compared to no boost (92.7% versus 84.9%, respectively). Patients <40 years old, positive margin status and high mitotic index were found to have significantly higher chance of local tumour recurrence [14].

## Discussion

Indications for boost therapy in patients after breast conservative therapy remain a controversial topic in current oncology practice. Boost therapy offers improved local control, possibly due to the elimination of microscopic tumour cells in the surgical field. In this article, we examined its effect on breast cancer patients through analysing seven studies published since 1997.

Whole breast irradiation therapy with or without boost is commonly performed on patients after BCS. Studies have consistently shown improvement in LRR rates, even with limited nodal involvement [17, 18]. Young patients with early stage breast cancer have worse prognosis than older patients in terms of local relapse [19, 20]. The results of the EORTC trial [14] are in line with those findings where the largest absolute benefit was found in patients <40 years old.

There are several techniques to achieve tumour bed boost after a lumpectomy and these may differ based on institutional practice. While the post-operative approach is most commonly employed, intra-operative surgical bed boost with 10–20 Gy prior to adjuvant whole breast radiation has been described and can be done using multiple platforms including low-kV energy X-ray sources, electrons on mobile linear accelerators [21–23].

Post-operatively, boost radiation therapy after whole breast radiation can also be delivered in several methods including the use of electrons or photons delivered by linear accelerator, or interstitial brachytherapy delivered by radioactive sources such as iridium [24]. Target volume localisation is often achieved by reviewing pre-operative imaging and delineating the corresponding area on the planning CT simulation. Some centres advocate obtaining second CT simulation in lateral decubitus position for planning [25]. Alternatively, some practices may utilise the surgical scar as a landmark and boost the corresponding breast tissue below that scar. Even when preoperative axial imaging is used, intra-operative placement of clips can aid in volume localisation, especially in the setting of oncoplastic reconstruction [26]. External beam radiation techniques are usually prescribed as 10–16 Gy in 1.8–2 Gy fractions. Photon boosts can be planned using reduced tangents or 3D conformal techniques, whereas appositional electron planning often entails selecting appropriate energies and prescribing the dose to the 90%–95% isodose line. Similar dosing regimens are agreed upon in both European (St. Gallen Consensus) [27] and American (NCCN) [11] guidelines. It remains unclear, however, whether the improved outcomes of such regimens would outweigh the adverse events of increased radiation exposure. Furthermore, a prospective phase II trial by Gupta *et al* [28] assessed the use of hypofractionated whole breast irradiation using 36.63 Gy delivered over 11 fractions, followed by a tumour bed boost of 13.32 Gy in four fractions. The study results showed over 95% locoregional control and OS at 5-year follow-up. The trial showed minimal toxicity from the following regimen, with favourable cosmetic outcomes, proving the feasibility of hypofractionation followed by radiation boost in this patient population [28]. Additionally, radiation oncologists have considered incorporating the tumour bed boost as part of whole breast irradiation. The following would serve patients well by saving them treatment time and cost. Moreover, the use of a simultaneous integrated boost has shown some dosimetric advantages in terms of target volumes and organs at risk [29].

Radiation therapy treatment for patients with breast cancer is generally well tolerated, with only minimal toxicity. Generally speaking, adverse events are divided into acute and chronic. Acute adverse effects include fatigue, sore throat, cutaneous fibrosis and dermatitis, which can develop during or right after treatment. On the other side, chronic side effects tend to develop months after treatment, and typically include radiation pneumonitis, secondary malignancies, radiation induced cardiotoxicity, hypothyroidism and arm lymphedema [15, 30]. Breast radiotherapy also poses a significant adverse effect on cosmesis and those planning to undergo immediate prosthetic breast reconstruction. A meta-analysis of 15 randomised controlled trials demonstrated an increased risk of overall complications (odds ratio (OR): 3.45, *p* < 0.00001) and capsular contracture (OR: 5.26, *p* < 0.00001) in patients undergoing prosthetic reconstruction [31]. An analysis of the ‘boost versus no boost’ trial showed increased long-term risk of fibrosis in patients receiving boost therapy (*p* < 0.01) [32].

**Figure 2.**
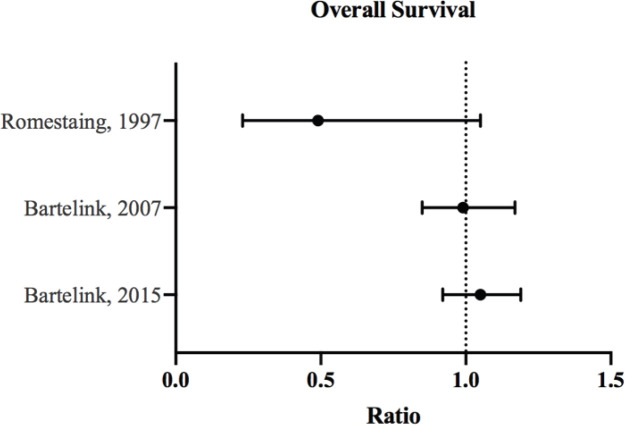
OS ratios and CI for the comparison of radiation therapy boost to no-boost. Bartelink *et al* [12, 13] analysed the same trial population at different follow-up periods, and both reported HRs with 95% and 99% CI, respectively. Romestaing *et al* [10] reported RR with 95% CI.

In all the published studies analysed, tumour bed boost did not offer significant improvement in OS (Figure 2). The effect on local control, however, was very evident [13, 14, 33, 34] (Figure 1). The following portrays a discrepancy between local control and OS benefit in patients receiving boost therapy. While it is possible that local control does not translate to improved survival, this inconsistency may be attributed to the good salvage options offered to recurrent cases.

The studies included in the literature review did not involve the use of neoadjuvant or targeted therapies such as trastuzumab. Trastuzumab showed significant evidence of improved disease-free survival and OS in human epidermal growth factor receptor 2 (HER-2) positive patients [35]. A retrospective analysis of the HERceptin Adjuvant trial aimed at analysing the role of radiation boost in patients with HER-2 positive breast cancer. All patients received trastuzumab, and underwent BCS. At 11-years follow-up, the study showed no difference in local control between patients that underwent tumour boost, and those that had whole breast irradiation without boost [36]. As such, the increased effectiveness of current systemic therapy regimens significantly undermines the applicability of the old trials to the current era of breast cancer treatment.

Previous studies focused on cellular and demographic stratification of breast cancer patient groups (mostly by age) to identify those most likely to benefit from boost radiotherapy. Recent advances in molecular profiling of breast cancer may add an extra layer of stratification to be examined in future studies and possibly help guide clinicians for better individually tailored radiation therapy decisions based on locoregional risk stratification and radio-sensitivity of each cancer subtype. For example, hormone receptor negativity and HER-2 receptor positivity were associated with increased risk of LRR and no improvement in OS when treated with post-mastectomy radiation therapy [37]. Patients in this study, however, did not receive anti-HER-2 treatment, and this makes it difficult to extrapolate the impact of boost therapy in the contemporary systemic therapy era. Others have also proposed using a 21-gene recurrence score assay to predict the benefit of radiotherapy in grey zone scenarios [38]. Such approaches can be considered in future selection of patients for boost radiotherapy.

An ongoing clinical trial, Young boost trial, aims to address these considerations among others. The effects of an increased radiation boost dose, 26 Gy versus 16 Gy, on LRR will be investigated at 10 years follow-up. This is of importance in younger patients (<50 years), who have higher risk of LRR, even after boost therapy [12]. The second aim is to analyse different genetic or protein profiles that may correlate with LRR, distant metastasis, survival and radiosensitivity.

A recent population-based study in the Netherlands demonstrated surprisingly low rate of LRRs, as low as 1.7% at 10 years in particular subtypes [39]. This can be related to the combination of improvements in diagnostic as well as local and systemic therapeutic improvements. As the impact on cosmetic outcomes after especially a high boost dose is very significant [40], a critical appraisal of the indication for any boost is required, even for younger patients.

Boost radiation delivery varies considerably from one country to another, from extremely frequent in countries where reimbursement/payment is based on a per-fraction principle to around 45% in The Netherlands and only 15% in Denmark [41].

Our current review presents qualitative research findings without systematic data analysis. Therefore, the findings presented should be considered in the light of some limitations. Furthermore, risk of bias was not assessed in this study. However, we present a review covering all major studies that addressed the benefits of boost in breast cancer treatment.

Due to the insufficiency of up to date high level evidence and in the light of stated limitations of this article, we recommend that treatment decisions for the administration of surgical cavity boost be based on individual clinicopathological factors associated with significant risks of LR. These include age, surgical margins, tumour grade and high lymphatic involvement. Those patients are most likely to derive benefit. It is equally important to compare the overall beneficial effects of treatment to the associated increased severity of side effects associated with higher doses of radiation therapy. With the rapid progress of targeted therapies and neoadjuvant regimens, the added benefit of cavity boost needs to be carefully studied in future clinical trials.

## Conclusion

In conclusion, data from previous studies have shown improvement in local control in patients undergoing surgical cavity boost after breast conservative surgery. However, this intervention was not demonstrated to improve OS. With the advent of targeted therapy and more efficacious chemotherapeutic regimens, the added value of extra local radiation dose needs to be evaluated. Future studies, including the analysis of real-life data and randomised trials are still needed to better assess the role of boost therapy after breast conservative surgery. Further focus on comparing different molecular and genetic subgroups of breast cancer patients might lead to more accurate results and better individualised radiotherapy decisions in the modern era.

## Compliance with ethical standards

### Funding

None.

### Conflicts of interest

The authors declare that they have no conflicts of interest.

### Ethical approval

This article does not contain any studies with human participants performed by any of the authors.

## Authors’ contributions

1. Majd Kayali: conceptualisation, data curation, formal analysis, writing – original draft, writing – review and editing.
2. Joseph Abi Jaoude: conceptualisation, data curation, formal analysis, writing – original draft, writing – review and editing.
3. Paul Ramia: formal analysis, writing – original draft, writing – review and editing.
4. Hazem Assi: formal analysis, writing – original draft, writing – review & editing.
5. Fady Geara: formal analysis, writing – original draft, writing – review & editing.
6. Philip Poortmans: formal analysis, supervision, writing – original draft, Writing – review and editing.
7. Youssef H Zeidan: conceptualisation, formal analysis, supervision, validation, writing – original draft, writing – review and editing.

## Figures and Tables

**Table 1.** Clinical outcomes of tumour-bed boost radiation therapy.

Study ID	Number of patients	Median follow-up (years)	Inclusion criteria	Surgery type	Systemic therapy	Boost versus no Boost	Boost dose	LR (%)	Ratio	CI	*p*	OS (%)	Ratio	CI	*p*
Moran *et al* [34]	4,131	9	DCIS	BCS	CT and/or HT	Boost: 2,661 No boost: 1,470	14 Gy	8.4 12	HR: 0.73	95% CI: 0.57–0.94	0.01				
Bartelink *et al* [12]	5,318	17.2	T1–2, N0-1, M0	Lumpectomy, complete resection, axillary dissection	CT and/or HT	Boost: 2,661 No boost: 2,657	16 Gy	9 13				59.7 61.1	HR: 1.05	99% CI 0.92–1.19	0.323
Bartelink *et al* [13]	-	10.8	-	-	-	Boost: 2,661 No boost: 2,657	-	6.2 10.2	HR: 0.59	99% CI: 0.46–0.76	<0.0001	80.4 80.4	HR: 0.99	95% CI: 0.85–1.17	0.935
Bartelink *et al* [33]	-	5.1	-	-	-	Boost: 2,661 No boost: 2,657	-	4.3 7.3	HR: 0.59	99% CI: 0.43–0.81	<0.001	91 87			0.63
Millar *et al* [42]	498	7	Invasive carcinoma	Local excision, axillary sentinel node biopsy or clearance	CT and/or HT	Boost: 247 No boost: 251	16 Gy		HR: 1.2		0.599				
Romestaing *et al* [10]	1,024	3.3	Ductal carcinoma, ≤3 cm	Tumourectomy or quadrantectomy, free margins, axillary LN resection	CT and/or HT	Boost: 521 No boost: 503	10 Gy	3.6 4.5	RR: 0.34	95% CI: 0.12–0.95	0.044	92.9 90.4	RR: 0.49	95% CI: 0.23–1.05	0.24
Polgar *et al* [14]	207	5.3	T1–2, N0–1	BCS, free margins, axillary dissection	CT and/or HT	Boost: 104 No boost: 103	16 Gy	6.7 15.5	RR: 0.42	95% CI: 0.17–1.02	0.049				
